# Food allergy and anaphylaxis – 2065. Food-dependent exercise-induced anaphylaxis in a patient with sensitization to pork

**DOI:** 10.1186/1939-4551-6-S1-P148

**Published:** 2013-04-23

**Authors:** Hyun Hee Lee, Kyung Eun Lee, Kyu-Earn Kim

**Affiliations:** 1 Department of Pediatrics, Kwandong University College of Medicine, Goyang, South Korea; 2 Department of Pediatrics and Institute of Allergy, Yonsei University College of Medicine, Seoul, South Korea; 3 Yonsei University College of Medicine, South Korea

## Background

Food-dependent exercise-induced anaphylaxis (FDEIA) is a specific variant of exercise-induced anaphylaxis that requires both vigorous physical activity and the ingestion of specific foods. In particular, it is rare that FDEIA is associated with meat in Korea.

## Methods

A 15-year-old female presented with generalised urticaria, dyspnea, severe cough, headache, dizziness, and vomiting after singing and dancing for 1 hour and after ingesting grilled pork. Serum specific IgE and skin prick tests were performed. Oral food challenge with boiled pork meat were done. We started boiled pork meat 10g to 160g every 30 mimutes. Exercise provocation test before and after pork ingestion were done.We did IgE immunoblotting with patient's serum by cooked(grilled, boiled) and raw pork allergen.

## Results

Skin prick tests showed a strong positive reaction to pork, whereas the results of the oral food challenge and exercise provocation tests were negative. However, the exercise provocation test after pork ingestion showed a positive reaction manifested by generalized urticaria, cough, mild dyspnea, and 23% decreased peak expiratory flow rate. In IgE immunoblotting, 3 allergens of pork (67 kDa, 90 kDa, and 15 kDa) reacted with patient's serum by grilled pork allergen.

## Conclusion

We report a case of pork-dependent exercise-induced anaphylaxis in a patient with sensitzation to pork. A patient was instructed to avoid exercise after pork meat ingestion in the future.

